# Retinal Image Matching Using Hierarchical Vascular Features

**DOI:** 10.1155/2011/749054

**Published:** 2011-10-13

**Authors:** Alauddin Bhuiyan, Ecosse Lamoureux, Baikunth Nath, Kotagiri Ramamohanarao, Tien Y. Wong

**Affiliations:** ^1^Department of Computer Science and Software Engineering, The University of Melbourne, Melbourne, VIC 3010, Australia; ^2^Centre for Eye Research Australia, The University of Melbourne, Melbourne, VIC 3002, Australia

## Abstract

We propose a method for retinal image matching that can be used in image matching for person identification or patient longitudinal study. Vascular invariant features are extracted from the retinal image, and a feature vector is constructed for each of the vessel segments in the retinal blood vessels. The feature vectors are represented in a tree structure with maintaining the vessel segments actual hierarchical positions. Using these feature vectors, corresponding images are matched. The method identifies the same vessel in the corresponding images for comparing the desired feature(s). Initial results are encouraging and demonstrate that the proposed method is suitable for image matching and patient longitudinal study.

## 1. Introduction

 Recent advancement in retinal imaging enable us to use images for earlier diagnosis of diseases, image matching in biometric security application, and information retrieval purposes. Studies show that changes in the retinal vascular features such as vessel width, tortuosity, and branching angle are very important indicators for predicting hypertension and cardiovascular diseases [1, 2]. In addition, retinal vascular pattern is unique to each individual. Hence, a suitable approach that can accurately analyze retinal images for both disease diagnosis and image matching would be a very significant contribution in this image modality. 

**Figure 1:**
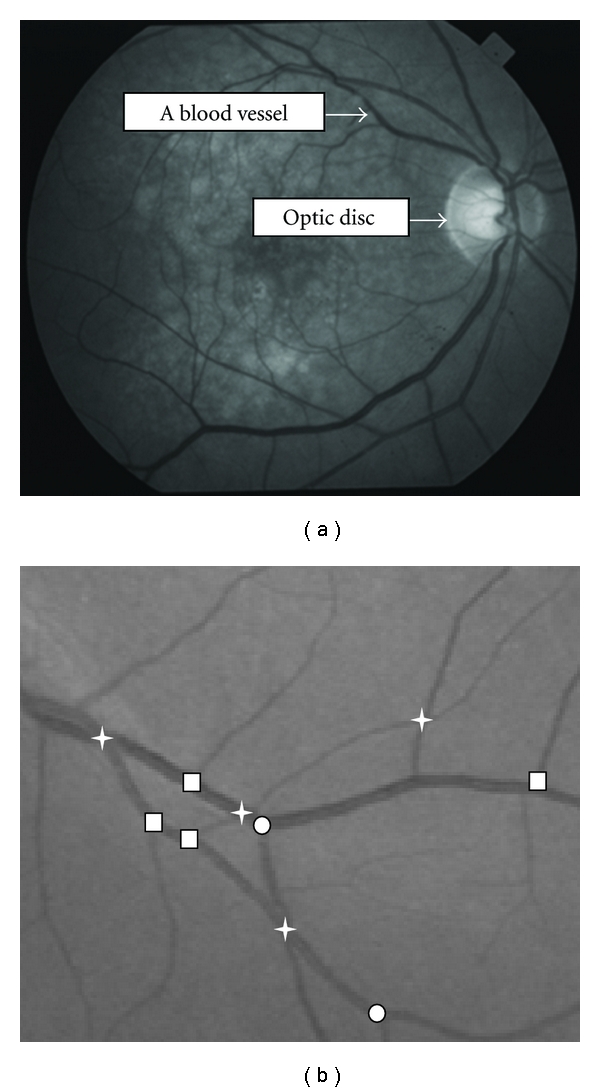
A retinal image showing the blood vessels (a) and a cropped section showing vascular Bifurcation (circle), branch (square) and crossover (star) points (b).

 For disease prediction or clinical trial, the most widely used approach is to take person's retinal image (Figure 1(a)) within a time interval and compare these images to observe the change(s) in the vascular features [3, 4]. In various studies [5, 6], authors have reported the effect of hypertension treatment on retinal vessel diameter and tortuosity performed on person's retinal images taken before and after medication. These studies considered mainly the manual or semiautomatic methods for image analysis that are very time consuming and expensive. Furthermore, these studies are based on analyzing a single feature which is not enough to observe multiple feature changes. None of these techniques is able to match the vascular features from two images based on vessel segments' hierarchical position, which is very important for automated patient longitudinal study. 

 Retinal image matching methods for person identification in the main register images after vessel segmentation, or match bifurcation or branch point in the corresponding images [7–9]. In the first approach, retinal blood vessels are used as the biometric parameter, with a prior registration stage needed to align the template image and the acquired image. The second approach segments the blood vessel from the image and computes the bifurcation/branch and crossover points (Figure 1(b)). 

 Vascular bifurcation/branch and crossover points are defined as follows. Vascular bifurcation split the vessel into two vessels. A branch is a new vessel formation where a minor (smaller width) vessel grows or comes out from a major (wider) vessel. A crossover is a region where two vessels (major or minor) cross each other. 

 Overall, bifurcation/branch and crossover points geometry should provide higher degree in generating unique pattern for an individual person. At present, retinal vascular bifurcation and branch points are considered as the same parameter for image matching applications. Classified bifurcation and branch points can add higher degree in the uniqueness of the retinal vascular pattern. Considering these issues, we propose an automatic retinal image matching method, which has high potential for patient longitudinal study and biometric security application. The method uses retinal vascular bifurcation, branch, and crossover points as features to match the images. We utilize the retinal vessel hierarchical property and construct a binary tree for each vessel to match images. 

 A number of vessel tree construction methods are available in the literature for retinal image [10–12]. However, none of them represents multiple features for each of the vessel segments hierarchically. Further, no method classifies the bifurcation and branch points to match the images which provides higher degree of uniqueness in the pattern. Our proposed method is able to match retinal images for authentication or analyzing multiple feature changes for disease diagnosis purposes. 

 The rest of the paper is organized as follows.  Section 2 describes the proposed method which includes the vascular tree model construction, invariant feature extraction, and tree matching based on the invariant features. Initial results on the matching method which can be used for patient longitudinal study are shown in Section 3.  Section 4 concludes the paper with planned future work.

## 2. Proposed Method

Our proposed image-matching method is based on the tree representation of the retinal vascular network which is a hierarchical representation of features of the vessel segments of blood vessels. In this paper, we mainly focus on the vascular invariant feature extraction, the feature vector construction, and the matching algorithm for patient longitudinal study. We use retinal bifurcation, branch, and crossover points to construct the feature vector. The vascular invariant features along with the vessel segments hierarchical position provide a unique pattern for each of the blood vessels in the retina for each person.

### 2.1. The Tree Model

**Figure 2:**
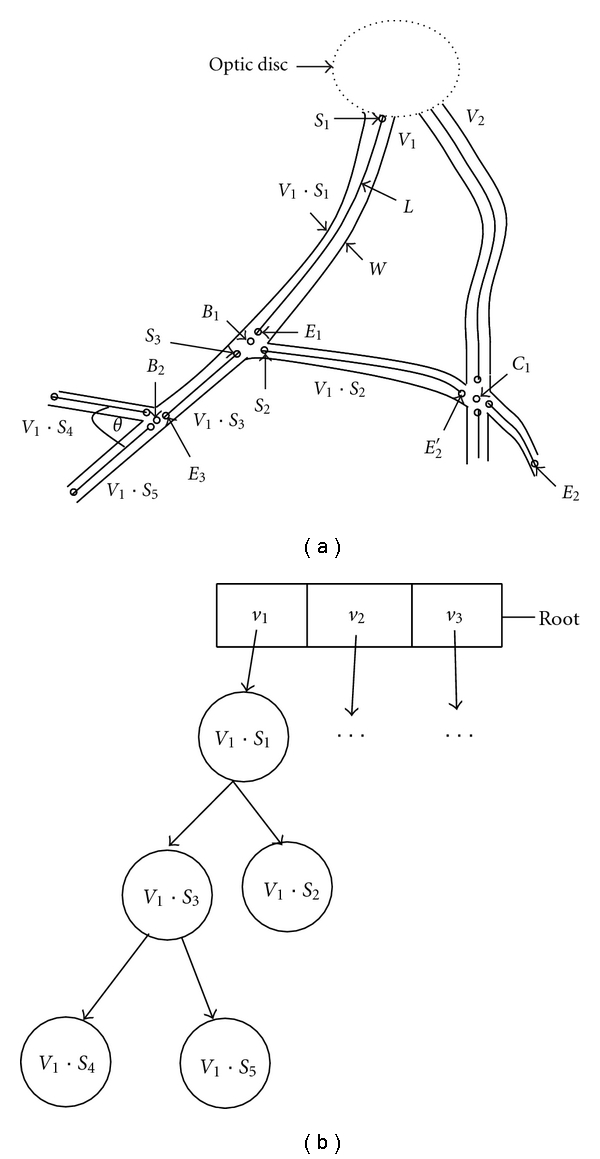
A schematic diagram of a retinal image showing two blood vessels (*V*_1_ and *V*_2_) and the optic disc (a) and the tree model representing the binary tree for vessel *V*_1_ (b).

We have applied the method for developing a tree structure (we call it tree model) of the retinal vascular network which is based on vessel centerline (*L*), width (*W*) and bifurcation (*B*_2_), and branch (*B*_1_) or crossover (*C*_1_) point information in the retinal optical fundus image as represented in Figure 2(a).

We have applied our previously reported methods for vessel segmentation and centerline detection [13] and bifurcation, branch, and crossover points detection [14] in the tree model. The construction of the tree model begins by tracking the vessel centerline from the border of the optic disc (OD) in the retinal image. This corresponds to the root node in the tree model. Each vessel originating from the optic disc is then represented by a binary tree and is linked to the root node in the tree model.

The blood vessel centerlines are fragmented into different vessel segments based on the bifurcation, branch, and crossover points. Initially, these landmark points and their corresponding vessels' start or end points are computed and stored [14]. Hence, a vessel segment starts from the optic disc boundary or a bifurcation or a branch point and ends at a bifurcation or branch point. We note that a vessel is split into two in any branch/bifurcation point. We construct a binary tree for each vessel, where each parent vessel segment has one or two children vessel segments. Thus, for each vessel we construct a binary tree which can be a complete or incomplete binary tree. We compute the position of the vessel segment and insert them as left and right children node for each parent.

**Figure 3:**
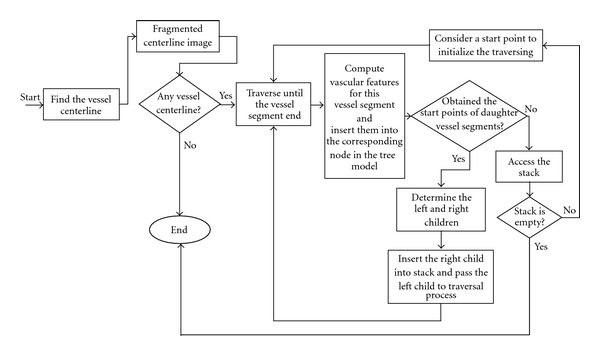
The flow diagram of the proposed retinal vascular network model (i.e., the tree model).

For each vessel segment, the features, are computed and inserted into its corresponding node in the tree model. This is shown in Figure 2 for vessel *V*_1_. Vessel segment *V*_1_ · *S*_1_ (starts from *S*_1_ and ends at *E*_1_) is traced, and its features are inserted into the corresponding node (node *V*_1_ · *S*_1_ in Figure 2(b)) in the tree model. Its daughter vessel segments *V*_1_ · *S*_2_ and *V*_1_ · *S*_3_ are then traced. These vessel segments' features are inserted into the tree model as children nodes of *V*_1_ · *S*_1_ (Figure 2(b)). Similarly, vessel segments *V*_1_ · *S*_4_ and *V*_1_ · *S*_5_ are inserted as the children node of *V*_1_ · *S*_3_. Generally, a vessel segment appears in two parts at a crossover point (*C*_1_ in Figure 2(a)). Therefore, the crossover point is used to trace the other part of a vessel segment. For example, as in Figure 2(a), when the crossover point *C*_1_ is encountered, the end point of the vessel segment *V*_1_ · *S*_2_ is measured as *E*_2_ instead of *E*_2_′. The tree model construction method is shown in Figure 3. 

### 2.2. Invariant Features for Image Matching

For image matching we consider the vascular invariant features that is, the features invariant to rotation, translation or scaling (where the scaling is equal in all dimensions). The features are: vessel segment's length to width ratio (*L/W*), bifurcation or branch point, information on crossover existence in a vessel segment, crossover location in the vessel segment and acute angle between the parent and smaller daughter vessel segments. These invariant features provide a unique pattern for any individual's retina. For each vessel segment, these feature values are inserted into the corresponding node in the tree model.

### 2.3. Vascular Feature Extraction

To extract vascular features we consider vessel centerline image, edge image, and fragmented centerline image. The methods for obtaining these images are described in [15].

**Length to Width Ratio (L/W)**

We implement region growing algorithm [16] to extract the length of a vessel segment. Using the optic disc (OD) center and radius, we search a circular region around the OD, and determine the starting pixel of a vessel centerline, and traverse through the center line until we reach its end point.

For each vessel segment, the starting pixel is considered for initiating the traversal process in the fragmented vessel centerline image. The traversal algorithm uses the 3 × 3 neighborhood connectivity check to find the neighboring pixels in the vessel segment. For this, a mask is applied by considering the starting pixel of any vessel segment as its center. Once a neighboring pixel is found, it replaces the previous center pixel in the mask. Each time a pixel position is considered, a flag value is assigned to this. Once the traversal process reaches the vessel segment's end point, it stops if it belongs to a bifurcation or a branch. The traversal process also stops if the vessel segment's end point is not obtained after a certain number of iterations. Once the end point is obtained, the total length of this vessel segment is returned in pixel number, and average width [15] is computed. These values are inserted into the corresponding node in the tree model.

**Bifurcation or Branch Point**

We classify each landmark as bifurcation or branch point (Bi/Br). The bifurcation, branch and crossover point classification method is applied by using the procedure described in [17]. We assign a unique value (1 for bifurcation and 0 for branch) in the feature value for each of the vessel segment.

**Existing Crossover**

For each vessel segment, if any crossover point exists we assign a unique value for the node. We insert 1 for presence and 0 for absence of the crossover point to the node value.

**Crossover Location**

For the vessel segment, if there is any existing crossover point, we assign the positional information of that crossover. For example, if the actual length is 100 pixels and the crossover position is on the 70th pixels from the starting of the vessel segment, we assign the ratio of its position to the total segment length, which is 0.7. If there is no crossover, we assign 0 for this feature value.

**Acute Angle between the Parent and Smaller Daughter Vessel**

**Figure 4:**
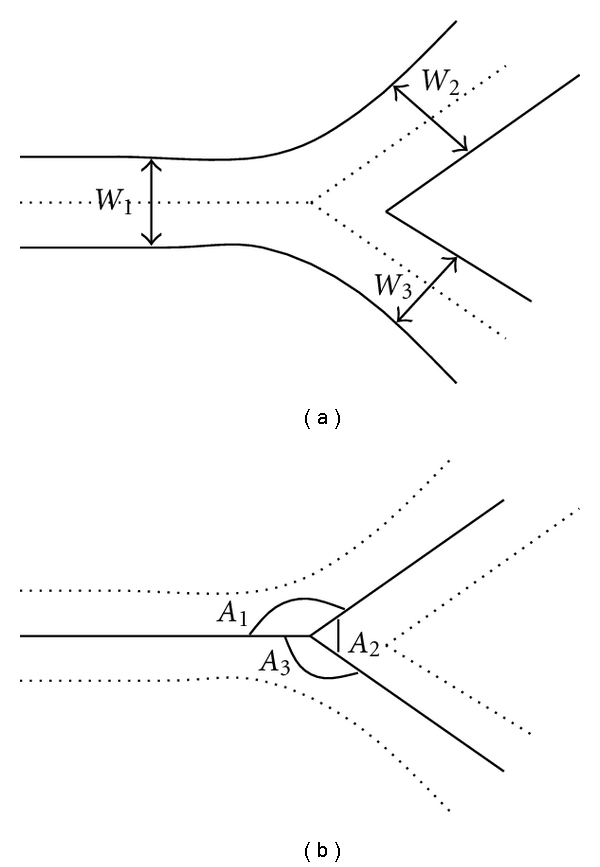
Vascular widths (a) and angles (b).

We find the acute angle between the parent and the smallest vessel. The parent and smallest (daughter) vessel is classified by using the width of each of the vessels for any bifurcation or branch point. The vessel segments for each bifurcation or branch point are sorted based on their width. Then, the angle between the centerlines of highest and lowest width vessels' are computed.  Figure 4 shows the vessel segments width and the corresponding angles.

Once we construct the binary trees for all the vessels, we normalize the angle and length-to-width ratio.

### 2.4. Accessing Vascular Features from the Tree Model

We access the vascular features from the tree model by using the preorder tree traversal algorithm [18]. To traverse a nonempty binary tree in preorder, we perform the following operations recursively at each node. Starting at the root of a binary tree, we retrieve the values for the current node (referred to as “visiting” the node) then traverse to the left child node, and finally traverse to the right child node.

### 2.5. Feature Vector Construction and Matching

Each binary tree is traversed, and the invariant feature values are obtained from each node (Table 1). Following this, for each node (i.e., each vessel segment) we construct a feature vector from these invariant feature values. We note that the invariant features with their actual hierarchical position in the tree model enhance the uniqueness of a feature vector. We then compare the corresponding feature vectors on the tree traversal order for finding the binary trees (which represent the same vessel) from the tree models.

For image matching, our goal is to find the corresponding binary trees in the tree model that represent the same vessel in the two retinal images. We perform the bitwise comparison of the feature vectors to find the distance between the corresponding binary trees. For each corresponding feature vector, if a match is found, we add zero to the final matching distance and one if there is no match. We note that the matching distance is the distance between the binary trees (i.e., the vessels) in node number. We obtained the matching matrix by comparing the corresponding binary trees in the tree models.

For finding the corresponding binary trees, we consider the upper two levels' nodes from each of the binary trees. This way we are able to obtain sufficient number of feature vectors for matching to find the corresponding binary trees from the tree models. This also makes the matching process faster and reduces computation complexity which is higher when considering more levels of nodes in the binary trees. Once a match is obtained, another binary tree is considered from the respective tree model to find the corresponding binary tree to the other tree model. This matching process continues until all the corresponding vessels are obtained.

## 3. Experimental Results

We match the tree models obtained from the retinal images of an individual. We trace the corresponding blood vessels to observe the changes in a particular period of time. We note that each vessel has a unique pattern on its feature vectors. Therefore, we are always able to find the corresponding vessel or binary tree in the tree models.  Table 2 shows the distance of different blood vessels in node number for an image.

To evaluate the performance of our matching method on the images taken at different conditions (e.g., second image may be rotated or shifted while capturing), we rotated the image from ±5° to ±30°. Unfortunately, we do not have the data on images taken at different times for the same person. Hence, we rotated the same image and constructed the tree model for each rotated image and obtained the distance matrix in node numbers for one vessel with the other vessels. This is done by comparing the first tree model which is obtained from the original image with the tree models constructed for the images at different rotating angles.  Table 3 shows the distance matrix for vessel 1 with the original tree model and the obtained tree model after rotating the image.

We observe that for smaller rotation angle, there is no difference on the feature vectors' attributes. With higher rotation angle, some changes are introduced in feature values such as vessel length, width, and crossover position. However, such errors are introduced due to the discretization of the pixels' actual position during rotation operation of the image. To overcome this problem, we consider a threshold value of ±5% for the length to width ratio and actual crossover position and received the distance as 0 for the same vessel, while the distances with other vessels remain the same.  Table 4 shows the distance matrix for vessel 1 after applying the thresholding technique. Using the tree models, we also compare the distances between the same vessels in the images with this technique.  Table 5 shows the distance matrix between the same vessels in the image (from the two tree models).

From these observations, we can conclude that the proposed vascular network model may be suitable for comparing vascular feature(s) between two images for patient longitudinal study. Our main focus has been to find the corresponding vessel, that is, the binary tree from the tree models. In worst case, if the minimum number of nodes are not matched from the corresponding binary trees, we can rely on the best match between the binary trees assuming that we are matching the tree models for the same person's images. We can, therefore, decide about the corresponding binary trees. To evaluate the robustness of our method for image matching, we considered 20 images in the STARE database. For each image, the tree model is constructed, and then, the nodes are compared to find the distance matrix.  Table 6 shows the distance in node number between the tree models for ten (randomly selected) images.

## 4. Conclusions

In this paper, we described a novel approach for retinal image matching which is highly suitable for patient longitudinal study. Initial results suggest that the method is very accurate in matching the retinal images and finding the corresponding blood vessels. Based on this method, the medical practitioners can observe changes on different vascular features for each of the vessel segments. Simple modification of the method can also be suitable for biometric security application. At present, we are acquiring multiple images for the same person to enable a large-scale study and further validation of the method. We aim to perform two case studies in biometrics and patient longitudinal study using our proposed retinal image-matching algorithm.

## Figures and Tables

**Table 1:** The invariant features for vessel segments in the nodes of a tree model.

Nodes	Invariant features				
	*L/W*	Bi/Br	Crossover	Cross pos.	Acute angle
1	88.77	1	0	0	0.877
2	18.27	0	0	0	1.126
3	43.12	0	0	0	0
4	34.27	1	1	0.79	0.788
5	53.42	1	0	0	0.689

**Table 2:** The distance matrix between the vessels for an image.

	Distance between the vessels in node number				
	Vessel 1	Vessel 2	Vessel 3	Vessel 4	Vessel 5
Vessel 1	0	7	7	7	7
Vessel 2	7	0	7	7	7
Vessel 3	7	7	0	7	7
Vessel 4	7	7	7	0	7
Vessel 5	7	7	7	7	0

**Table 3:** The distance matrix with vessel 1 for different rotating angles.

Rotation	Distance with vessel 1 in node number				
	Vessel 1	Vessel 2	Vessel 3	Vessel 4	Vessel 5
0°	0	7	7	7	7
5°	0	7	7	7	7
10°	0	7	7	7	7
15°	2	7	7	7	7
20°	2	7	7	7	7
25°	4	7	7	7	7
30°	4	7	7	7	7

**Table 4:** The distance matrix after applying the thresholding with vessel 1 for different rotating angles.

Rotation	Distance with vessel 1 in node number				
	Vessel 1	Vessel 2	Vessel 3	Vessel 4	Vessel 5
0°	0	7	7	7	7
5°	0	7	7	7	7
10°	0	7	7	7	7
15°	0	7	7	7	7
20°	0	7	7	7	7
25°	0	7	7	7	7
30°	0	7	7	7	7

**Table 5:** The distance between the same vessel represented by the two different tree models after applying the thresholding.

	Distance between				
Rotation	Vessel 1	Vessel 2	Vessel 3	Vessel 4	Vessel 5
	to	to	to	to	to
	Vessel 1	Vessel 2	Vessel 3	Vessel 4	Vessel 5
0°	0	0	0	0	0
5°	0	0	0	0	0
10°	0	0	0	0	0
15°	0	0	0	0	0
20°	0	0	0	0	0
25°	0	0	0	0	0
30°	0	0	0	0	0

**Table 6:** The distance matrix between the images.

	The distance between the images in node number									
	One	Two	Three	Four	Five	Six	Seven	Eight	Nine	Ten
One	0	31	42	34	47	29	56	48	49	51
Two	31	0	43	35	48	30	57	49	50	52
Three	42	43	0	46	59	41	68	60	61	63
Four	34	35	46	0	51	33	60	52	53	55
Five	47	48	49	51	0	46	73	65	66	68
Six	29	30	41	33	46	0	55	47	48	50
Seven	56	57	68	60	73	55	0	74	75	77
Eight	48	49	60	52	65	47	74	0	67	69
Nine	49	50	61	53	66	48	75	67	0	70
Ten	51	52	63	55	68	50	77	69	70	0
